# Baseline-Dependent Immunometabolic Responses During Prolonged Intermittent Fasting: A Secondary Integrative Analysis

**DOI:** 10.3390/nu18121954

**Published:** 2026-06-17

**Authors:** Zulrahman Erlangga, Samaneh Souita, Imad Hamdan, Yurdagül Zopf, Christoph Gutenbrunner, Abdulhadi Suwandi, Boya Nugraha

**Affiliations:** 1 Department of Internal Medicine, Krankenhaus Winsen, 21423 Winsen, Germany; 2 Department of Kidney, Liver and Metabolic Diseases, Children Hospital, Medizinische Hochschule Hannover, 30625 Hanover, Germany; 3 Department of Dermatology, Johannes Wesling Medical Centre, 32429 Minden, Germany; 4 Department of Rehabilitation and Sport Medicine, Medizinische Hochschule Hannover, 30625 Hanover, Germany; 5 Department of Medicine 1–Gastroenterology, Pneumology and Endocrinology, University Hospital Erlangen, Friedrich-Alexander University Erlangen-Nürnberg, 91054 Erlangen, Germany; yurdaguel.zopf@uk-erlangen.de; 6 Hector-Center for Nutrition, Exercise and Sports, Friedrich-Alexander University Erlangen-Nürnberg, 91054 Erlangen, Germany; 7 Deutsches Zentrum Immuntherapie, University Hospital Erlangen, Friedrich-Alexander University Erlangen-Nürnberg, 91054 Erlangen, Germany; 8 Hannover Rehabilitation Service & Science Consulting, 30655 Hannover, Germany

**Keywords:** intermittent fasting, immunometabolic, autophagy, inflammasome

## Abstract

**Background:** Prolonged intermittent fasting is associated with metabolic and immune adaptation; however, the extent to which transcriptional immune responses translate into systemic inflammatory changes, and how these processes relate to autophagy, senescence-associated signaling, and inflammasome regulation, remains incompletely understood. **Methods:** This study represents a secondary integrative analysis of a previously characterized cohort of healthy young men undergoing Ramadan fasting. Longitudinal data across four time points (T1–T4) were re-analyzed, integrating targeted mRNA profiling of autophagy-, senescence-, and inflammasome-related genes with circulating cytokines and clinical parameters. Baseline-stratified regression and exploratory clustering were applied to assess inter-individual variability. **Results:** Fasting was associated with modest reductions in body weight (−1.78 ± 1.44 kg, FDR < 0.001) and BMI (−0.56 ± 0.47 kg/m^2^, FDR < 0.001), without hemodynamic instability. Autophagy-related transcripts (ULK1, ATG5) were upregulated, while senescence markers showed divergent regulation (p53↑, p21↓). Inflammasome-related genes (NLRP3, IL1B) increased at the transcriptional level; however, circulating IL-1β and IL-6 remained stable and TNFα decreased (FDR < 0.001), indicating dissociation between transcriptional priming and systemic cytokine output. ΔNLRP3 was inversely associated with baseline expression (β = −1.88, R^2^ = 0.31, *p* = 0.0056), suggesting baseline-dependent transcriptional responsiveness. Responses followed a continuous spectrum rather than discrete subtypes. **Conclusions:** Prolonged intermittent fasting is associated with coordinated immunometabolic remodeling characterized by transcriptional changes in autophagy-, senescence-, and inflammasome-related pathways, without systemic inflammatory escalation. Inflammasome-related responses appear baseline-dependent, suggesting graded immunological responsiveness rather than a uniform activation. These findings are hypothesis-generating and support the interpretation of fasting as a graded immunometabolic modulator rather than a uniform pro-inflammatory stimulus within the limitations of a secondary exploratory analysis.

## 1. Introduction

Prolonged intermittent fasting has long been practiced in various cultural and religious contexts and continues to attract scientific interest due to its biological and physiological effects [1,2]. Beyond weight reduction and metabolic modulation, fasting is increasingly recognized as a physiological stress model capable of activating fundamental cellular pathways involved in energy sensing, inflammation, and aging.

In our previously published work from this cohort of healthy young males, we demonstrated that 30 days of structured intermittent fasting induced measurable alterations in body composition, including reductions in body weight and adiposity, without overt clinical instability [3]. In a subsequent exploratory molecular analysis of the same cohort, we further observed modulation of genes related to autophagy, inflammasome signaling, and cellular senescence [4,5]. Collectively, these studies provided evidence of coordinated systemic and transcriptional adaptations during prolonged intermittent fasting, but primarily in a descriptive and pathway-focused manner.

However, important mechanistic and integrative questions remain unresolved. In particular, changes in gene expression do not necessarily translate into protein-level alterations or clinically meaningful inflammatory responses, especially in tightly regulated systems such as inflammasome signaling. Transcriptional upregulation of inflammasome-related genes such as NLRP3 or IL-1β may therefore reflect a priming state rather than full inflammatory activation [6]. Whether prolonged fasting induces functional inflammatory activation or represents a state of controlled transcriptional adaptation remains unclear and requires integration of molecular signals with circulating protein data and clinical phenotypes.

Autophagy regulators such as ULK1 and ATG5 play central roles in cellular quality control and metabolic flexibility during nutrient deprivation [7]. In contrast, senescence-associated pathways involving p53, p21, and p16 contribute to cell-cycle arrest and age-related tissue dysfunction [8]. The balance between autophagy activation and modulation of senescence-associated pathways may therefore determine whether metabolic stress results in adaptive remodeling or maladaptive cellular responses. Systemic cytokines such as TNFα, IL-6, and IL-1β further provide a functional readout of whether molecular signaling is translated into systemic inflammatory activation [9].

The present study represents a secondary integrative re-analysis of a previously well-characterized Ramadan fasting cohort in healthy young men [5,10,11]. While prior publications from this cohort focused on descriptive changes in body composition, metabolic parameters, and selected transcriptional or cytokine markers, the current analysis extends this work by providing a systems-level integration of longitudinal gene expression, circulating cytokine profiles, and clinical phenotypes within a unified analytical framework.

In addition, this study introduces a baseline-stratified analytical approach to investigate inter-individual variability in inflammasome-related transcriptional responses, with a particular focus on NLRP3 signaling dynamics. Importantly, the hypothesis that inflammasome-related responsiveness may be modulated by baseline expression levels was not prespecified, but emerged from exploratory analyses of the longitudinal dataset.

To address this, we combined longitudinal comparisons across four time points (T1–T4) with regression-based modeling to assess whether baseline immune state influences the magnitude of transcriptional adaptation during prolonged intermittent fasting. We hypothesized that prolonged intermittent fasting is associated with coordinated immunometabolic adaptations characterized by enhanced autophagy-related signaling and transcriptional changes in inflammatory pathways, without corresponding systemic inflammatory escalation, and that these responses may vary according to baseline immune status.

## 2. Materials and Methods

### 2.1. Ethics Approval

This study was approved by the Ethics Committee of Hannover Medical School (Ethics No. 6899; DRKS-ID: DRKS00008181) and conducted in accordance with the Declaration of Helsinki. Written informed consent was obtained from all participants. The study took place during Ramadan 2015 (June–July 2015).

### 2.2. Participants

Twenty-five healthy young adult males were enrolled, predominantly students and staff of Hannover Medical School. Eligible participants were ≥18 years old, free from acute or chronic medical conditions (including metabolic, cardiovascular, autoimmune, infectious, inflammatory, and psychiatric disorders), and committed to fasting from dawn to sunset throughout Ramadan [11].

Participants were excluded if they had relevant comorbidities, acute illness, or interrupted fasting for more than 7 days during the study period.

### 2.3. Study Design

This study represents a secondary molecular analysis of a previously published controlled Ramadan fasting cohort. The present work is an integrative re-analysis of an existing dataset, and no new experimental data were generated.

The analysis focused on longitudinal changes in gene expression related to autophagy (ATG5, BECN1, ULK1), inflammasome activation (ASC, NLRP3, IL-1β, TNFA), and cellular senescence (p16INK4A, p21, p53) during prolonged intermittent fasting [5].

Although the original study included both fasting and non-fasting participants, the current analyses were restricted to the fasting cohort, as complete longitudinal molecular and cytokine datasets across all time points were available only within this group. Therefore, all inferential analyses were conducted within-subject in the fasting group.

Assessments were performed at four predefined time points: 1 week before Ramadan (T1), Mid-Ramadan (T2), End of Ramadan (T3), and 1 week post-Ramadan (T4) as previously described [5,11]. The present analyses should be considered exploratory and hypothesis-generating, as they were not prespecified in the original study protocol. In particular, the analysis of baseline-dependent NLRP3 responsiveness was conducted as a post hoc exploratory evaluation of longitudinal gene expression patterns.

### 2.4. Blood Sampling

Peripheral venous blood was collected between 08:00 and 10:00 a.m. at each time point. Plasma and serum samples were processed and stored at −80 °C until analysis [5].

### 2.5. RNA Isolation

Total RNA was isolated from whole blood using TRIzol reagent (Invitrogen, Carlsbad, CA, USA) followed by column-based purification (Monarch RNA Cleanup Kit, NEB, Ipswich, MA, USA), as previously described [5]. Complementary DNA (cDNA) was synthesized from 1 μg RNA using ProtoScript^®^ II First Strand cDNA Synthesis Kit (NEB, MA, USA) according to the manufacturer’s instructions [5].

### 2.6. QPCR

Gene expression analysis was performed using the QuantStudio™ 6 Pro system (Thermo Fisher Scientific, Waltham, MA, USA). All reactions were run in duplicates using Luna Universal qPCR Master Mix (NEB, MA, USA). Relative mRNA expression was calculated using the 2^−ΔΔCt^ method with GAPDH as the internal control [5].

### 2.7. Detection of Cytokines

Circulating protein biomarkers included inflammatory cytokines (TNF-α, IL-1β, IL-6, IL-8, IL-10, and IL-12), neurotrophic and growth factors (β-NGF, BDNF, GDNF, and IGF-1), and MMP-9. Protein measurements were generated using previously established immunoassay platforms. β-NGF, BDNF, GDNF, TNF-α, IL-8, and MMP-9 were quantified using a customized Human Magnetic Luminex Assay (R&D Systems, Minneapolis, MN, USA). IGF-1 was measured using a Quantikine Human IGF-1 ELISA (R&D Systems). Additional inflammatory cytokines were quantified using enzyme-linked immunosorbent assays (ELISA) or the BD™ CBA Human Inflammatory Cytokines Kit (BD Biosciences, San Jose, CA, USA) and analyzed on a BD LSR II flow cytometer (BD Biosciences, Heidelberg, Germany), as previously described [10,12].

For the present secondary analysis, protein markers were selected from these previously generated datasets and integrated with gene-expression measurements. Detection limits were defined according to manufacturer specifications, and values below the detection threshold were handled consistently across all analyses.

### 2.8. Body Composition Parameters, Mood, and Health-Related Quality of Life

Body composition parameters, including body weight (BW), body fat mass (BFM), body fat percentage (BFP), fat-free mass (FFM), body water mass (BWM), skeletal muscle mass (SMM), and estimated basal metabolic rate (BMR), were measured by InBody 230 (Model MW160, Inbody Co., Ltd., Seoul, Republic of Korea). All of these parameters were measured in the morning of TP1 to TP4 [10,11].

### 2.9. Statistical Analysis

All statistical analyses were performed using R (R Foundation for Statistical Computing, Vienna, Austria; version 4.5.2) within the RStudio environment (Posit Software, Boston, MA, USA; version 2026.01.0). Figures were generated using the ggplot2 (version 4.0.1) and pheatmap (version 1.0.13) packages.

Continuous variables are presented as mean ± standard deviation (SD), and categorical variables as counts and percentages. For this secondary molecular analysis of a previously published cohort, all longitudinal and regression analyses were restricted to the fasting cohort (*n* = 25), as complete longitudinal molecular and cytokine data across all time points were available only within this cohort.

The primary endpoint was defined as the change from baseline (T1) to the end-of-fasting time point (T3; approximately 30 days), corresponding to the main biological question of adaptive remodeling after sustained fasting exposure. Accordingly, changes were calculated as Δ(T3–T1), and paired comparisons were performed using paired *t*-tests. Effect sizes (mean differences for paired comparisons and regression coefficients for linear models) with corresponding 95% confidence intervals were reported where applicable.

Although measurements were obtained at four time points (T1–T4), intermediate (T2) and follow-up (T4) data were analyzed descriptively and in sensitivity analyses. Restricting primary inference to the T1-T3 contrast was performed to minimize multiple testing burden and reduce model overfitting in this exploratory dataset. T2 and T3 were therefore not included in primary inferential testing and were used exclusively to assess temporal dynamics and reversibility of observed responses.

Baseline characteristics between fasting and non-fasting participants were compared descriptively using independent two-sample *t*-tests (Welch’s correction as implemented in R) for continuous variables and Fisher’s exact test for categorical variables to confirm cohort comparability. No longitudinal or molecular comparisons were performed in the non-fasting group due to incomplete availability of matched repeated molecular measurements.

False discovery rate (FDR) correction was applied using the Benjamini–Hochberg procedure within predefined variable sets (clinical parameters, mRNA transcripts, and circulating protein markers). Adjusted *p*-values (FDR) < 0.05 were considered statistically significant. All statistical tests were two-sided.

Exploratory hierarchical clustering was performed within the fasting group using Δ(T3–T1) values of selected mRNA transcripts and circulating protein markers. Data were standardized using row-wise z-score normalization. Euclidean distance was computed and hierarchical clustering was performed using Ward’s minimum variance method (ward.D2). Participants were partitioned into two clusters (k = 2), with k selected based on dendrogram structure and exploratory interpretability. Given the modest cluster separation, clustering results were interpreted as exploratory and descriptive only. Only participants with complete Δ(T3–T1) data across all included variables were entered into clustering analyses.

Missing data were handled using complete-case analysis for each statistical test. No imputation was performed due to the exploratory nature of the study and small sample size. To assess baseline-dependent modulation of inflammasome-related responses, linear regression models were constructed with ΔNLRP3 as the dependent variable. Baseline NLRP3 expression and cluster membership were evaluated as predictors, with additional adjustment for skeletal muscle mass where indicated. To address potential regression-to-the-mean effects, additional sensitivity analyses were performed using an analysis of covariance (ANCOVA) framework, with T3 NLRP3 expression as the dependent variable and baseline (T1) NLRP3 expression as a covariate.

No formal sample size calculation or statistical power analysis was performed, as this study represents a secondary exploratory re-analysis of an existing dataset.

### 2.10. Use of Artificial Intelligence Tools

Artificial intelligence (AI)-based language tools (ChatGPT, OpenAI, San Francisco, CA, USA; GPT-5.5) were used to assist in language editing and structural refinement of the manuscript text. No AI tools were used for data generation, statistical computation, or independent data interpretation. All analyses were performed by the authors, who assume full responsibility for the accuracy and integrity of the work.

## 3. Results

### 3.1. Study Population and Baseline Characteristics

A total of 50 healthy male participants were included, comprising 25 individuals in the fasting group and 25 in the non-fasting group. Baseline demographic and anthropometric characteristics are summarized in Table 1.

There were no significant differences between groups with respect to age (26.1 ± 4.9 vs. 26.2 ± 4.9 years, *p* > 0.9), BMI (24.8 ± 3.6 vs. 24.6 ± 3.9 kg/m^2^, *p* = 0.8), body weight (77.8 ± 12.3 vs. 76.2 ± 21.4 kg, *p* = 0.7), body fat mass (16.7 ± 7.4 vs. 17.4 ± 8.4 kg, *p* = 0.7), or skeletal muscle mass (34.7 ± 5.1 vs. 34.6 ± 7.4 kg, *p* > 0.9).

However, ethnic distribution differed significantly between groups (*p* < 0.001). The fasting group comprised a higher proportion of participants from the Middle East (72%), whereas the non-fasting group included greater proportions of Asian (36%) and European (44%) participants. This imbalance reflects recruitment characteristics of the original cohort. Given the study’s within-subject longitudinal design for molecular analyses, no adjustment for ethnicity was applied in downstream analyses; however, this imbalance is considered in the limitations section.

Overall, aside from ethnic distribution, baseline demographic and anthropometric characteristics were comparable between groups (Table 1).

### 3.2. Clinical and Anthropometric Changes During Fasting

Within the fasting group (*n* = 25), body weight and BMI decreased after approximately 30 days of fasting (T3 compared with T1) (Table 2).

Body weight decreased by −1.78 ± 1.44 kg (FDR < 0.001), accompanied by a reduction in BMI of −0.56 ± 0.47 kg/m^2^ (95% CI −0.75 to −0.36, FDR < 0.001). Skeletal muscle mass declined modestly (−0.72 ± 1.22 kg; FDR = 0.018), as did fat-free mass (−1.07 ± 1.93 kg; FDR = 0.021).

Changes in body water mass (−2.86 ± 7.52 kg; FDR = 0.112), systolic blood pressure (−3.56 ± 10.55 mmHg; FDR = 0.139), diastolic blood pressure (0.84 ± 9.72 mmHg; FDR = 0.669), and body fat mass (0.64 ± 6.93 kg; FDR = 0.669) were not statistically significant after multiple-testing correction.

Collectively, these findings indicate that 30 days of fasting were associated with modest reductions in body weight and lean mass components, without significant changes in blood pressure or fat mass.

### 3.3. Transcriptional Responses to Fasting

**Figure 1:**
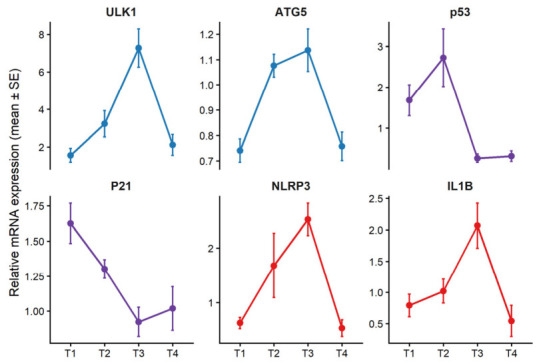
Longitudinal mRNA expression trajectories during Ramadan fasting in the fasting group (FG). Relative mRNA expression (mean ± SE) of ULK1, ATG5, p53, p21, NLRP3, and IL1B measured at four time points: 1 week before Ramadan (T1), mid-Ramadan (T2), end of Ramadan (T3; ~30 days), and 1 week post-Ramadan (T4) in the fasting group (*n* = 25). ULK1 and ATG5 showed increasing expression from T1 to T3 with partial return toward baseline at T4. p53 expression increased over time, whereas p21 expression decreased during fasting. NLRP3 and IL1B expression levels were elevated at T3 and decreased following fasting cessation.

Longitudinal analysis of mRNA expression demonstrated changes in autophagy-, senescence-, and inflammasome-related pathways during fasting (Figure 1). Autophagy-associated transcripts increased over the fasting period, with ULK1 and ATG5 showing progressive elevation from baseline (T1) to mid-fasting (T2) and reaching their highest levels at approximately 30 days (T3), followed by partial normalization one week after fasting (T4).

In parallel, p53 expression increased during fasting, reaching maximal levels at T3 before declining toward baseline at T4. In contrast, p21 expression decreased over the fasting period, reaching its lowest level at T3 and partially recovering thereafter. This pattern reflects differential regulation of senescence-associated transcripts during fasting and is consistent with adaptive stress-related transcriptional responses.

Inflammasome-related transcripts NLRP3 and IL1B also increased during fasting, peaking at T3 and declining after cessation, consistent with reversible transcriptional changes over time. Collectively, these findings indicate that prolonged fasting is associated with time-dependent changes in autophagy-, senescence-, and inflammasome-related gene expression.

Consistent with these trajectories, significant T1–T3 increases were observed for ULK1 (Δ = 5.71, 95% CI 3.38–8.05), ATG5 (Δ = 0.40, 95% CI 0.22–0.57), p53 (Δ = 4.50, 95% CI 2.54–6.47), NLRP3 (Δ = 1.90, 95% CI 1.20–2.60), and IL1B (Δ = 1.28, 95% CI 0.50–2.05), whereas p21 decreased (Δ = −0.70, 95% CI −1.02 to −0.38).

### 3.4. Circulating Cytokine and Protein Responses

**Figure 2:**
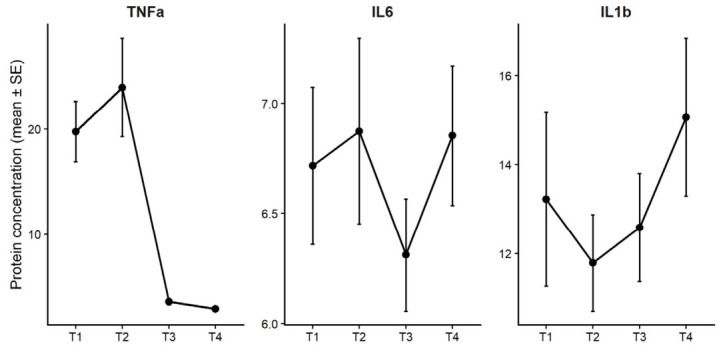
Circulating pro-inflammatory cytokine trajectories during Ramadan fasting in the fasting group (FG). Serum concentrations (mean ± SE) of TNFα, IL-6, and IL-1β measured at four predefined time points: 1 week before Ramadan (T1), mid-Ramadan (T2), end of Ramadan (T3; ~30 days of fasting), and 1 week post-Ramadan (T4) in the fasting group (n = 25). TNFα concentrations decreased over the fasting period, whereas IL-6 and IL-1β levels showed no consistent changes across time points.

Circulating pro-inflammatory cytokines did not demonstrate consistent increases across the fasting period (Figure 2). IL-6 and IL-1β protein levels remained largely stable across time points and were not significantly altered after FDR correction. In contrast, TNFα protein levels decreased during fasting (FDR < 0.001), with the reduction most evident at T3.

Together, these findings indicate a lack of concordant changes between transcriptional and circulating protein levels of inflammatory markers, suggesting a dissociation between mRNA expression and systemic cytokine concentrations during fasting.

### 3.5. Integrated Analysis of Transcriptional and Circulating Protein Responses

**Figure 3:**
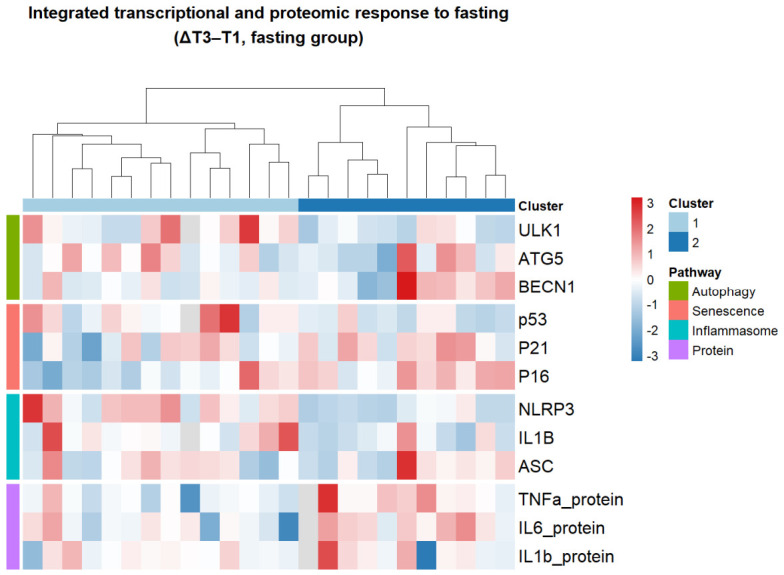
Integrated heatmap of Δ(T3–T1) molecular responses during fasting. Row-wise z-scored Δ(T3–T1) values of autophagy, senescence, inflammasome transcripts, as well as circulating cytokine proteins, in the fasting group (*n* = 25). Two participants contained missing molecular measurements and were therefore excluded from hierarchical clustering, leaving 23 participants for clustering analysis. Hierarchical clustering was performed using Euclidean distance and Ward’s method. Two exploratory clusters were identified (Cluster 1, *n* = 17; Cluster 2, *n* = 6). Cluster separation was limited (mean silhouette width = 0.12), indicating weak evidence for discrete molecular response subgroups.

To integrate molecular and circulating responses, standardized Δ(T3–T1) values were visualized using an integrated heatmap (Figure 3). This analysis showed increases in autophagy-related genes across participants in the fasting cohort. Senescence-associated markers displayed a variable pattern, with increased p53 expression alongside reduced p21 levels. Inflammasome-related transcripts (NLRP3 and IL1B) exhibited inter-individual variability across participants.

Notably, increases in NLRP3 and IL1B expression were not accompanied by corresponding changes in circulating IL-1β or IL-6 protein levels.

### 3.6. Baseline-Dependent Modulation of NLRP3

**Figure 4:**
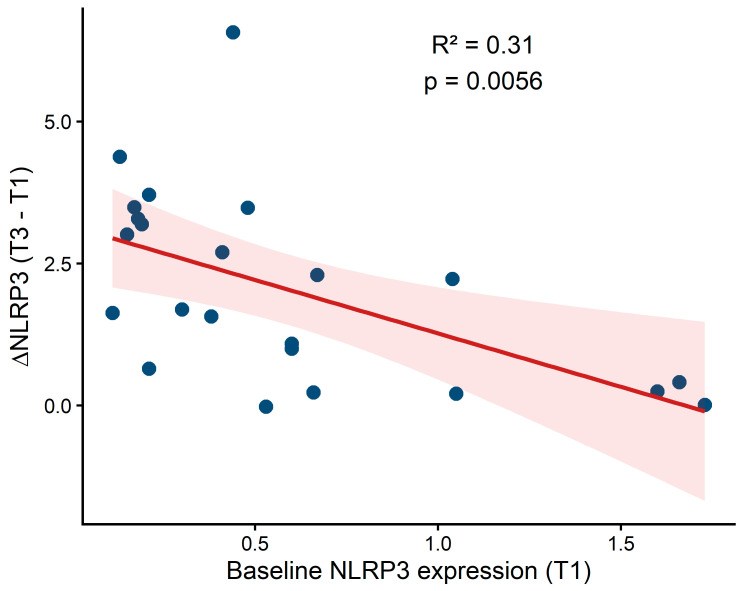
Association between NLRP3 expression and its change during fasting (*n* = 25). Scatter plot showing the relationship between baseline NLRP3 expression (T1) and ΔNLRP3 (T3–T1) in the fasting group (*n* = 25). Baseline NLRP3 expression was inversely associated with its change during fasting (β = −1.88, 95% CI −3.15 to −0.61, R^2^ = 0.31, *p* = 0.0056), with higher baseline levels associated with smaller increases over time.

To further investigate determinants of inter-individual variability, linear regression analysis showed that ΔNLRP3 was inversely associated with baseline NLRP3 expression (β = −1.88, 95% CI −3.15 to −0.61, R^2^ = 0.31, *p* = 0.0056) (Figure 4). Individuals with higher baseline expression exhibited smaller transcriptional increases during fasting, consistent with a baseline-dependent association between initial expression level and change over time.

In univariable analyses, skeletal muscle mass at baseline was also inversely associated with ΔNLRP3 (β = −0.14, *p* = 0.038). However, in multivariable models including both baseline NLRP3 expression and skeletal muscle mass, only baseline NLRP3 remained statistically associated with ΔNLRP3 (*p* = 0.015), whereas skeletal muscle mass was no longer statistically significant (*p* = 0.102). The combined model explained approximately 40% of the variance in ΔNLRP3.

To evaluate the robustness of this finding and account for potential regression-to-the-mean effects, an ANCOVA sensitivity analysis was performed using T3 NLRP3 expression as the dependent variable and baseline (T1) NLRP3 expression as a covariate. In this model, the associations did not reach statistical significance (β = −0.88, 95% CI −3.15 to 0.61, *p* = 0.164). However, the direction of effect remained consistent with the primary analysis.

Given that change scores inherently depend on baseline values, these findings should be interpreted with caution and may partially reflect regression-to-the mean effects rather than purely biological effects.

Overall, these results are consistent with a potential baseline-dependent pattern of transcriptional responsiveness. In this context, ‘baseline-dependent responsiveness’ refers strictly to the statistical association between baseline expression and longitudinal change, without implying mechanistic recalibration.

### 3.7. Exploratory Clustering Analysis

Exploratory hierarchical clustering of standardized Δ(T3–T1) molecular responses identified a two-cluster solution; however, cluster separation was weak (mean silhouette width = 0.12; Supplementary Figure S2), indicating limited evidence for robust cluster structure. Cluster-stratified analyses showed only minor differences in selected transcripts, and cluster membership did not independently predict ΔNLRP3 after adjustment for baseline expression.

Overall, these findings are consistent with a continuous distribution of inter-individual variability rather than discrete molecular response subgroups and should be interpreted as exploratory and hypothesis-generating only.

## 4. Discussion

In this integrated secondary molecular analysis of prolonged fasting in healthy young men, we observed coordinated immunometabolic changes characterized by activation of autophagy-related transcripts, differential modulation of senescence- and stress-associated signaling pathways, and inflammasome-related transcriptional changes consistent with a primed-like state at the mRNA level, without evidence of systemic inflammatory escalation. Importantly, these molecular adaptations occurred in the context of modest yet clinically tolerable anthropometric changes and stable hemodynamic parameters.

### 4.1. Autophagy Activation and Adaptive Stress Signaling

Consistent with our previous publications from this Ramadan cohort, fasting was associated with upregulation of autophagy-related genes, particularly ULK1 and ATG5 [5,10]. Early work from this cohort demonstrated that intermittent fasting enhances autophagy-associated transcriptional programs and promotes metabolic flexibility without inducing overt inflammatory activation. The present integrated analysis extends these findings by placing autophagy activation within a broader framework of coordinated stress-response signaling and immune modulation.

The observed increase in p53 expression alongside a reduction in p21 suggests a pattern consistent with adaptive stress signaling rather than senescence induction. Canonical senescence is typically characterized by coordinated activation of p53-p21 pathways and stable cell-cycle arrest, whereas the divergent regulation observed here may reflect transient and reversible stress responses. This interpretation is further supported by the overall absence of systemic inflammatory activation and the reversibility of transcriptional changes following cessation of fasting [13].

Taken together, these findings are consistent with fasting acting as a physiological metabolic stressor that activates cellular maintenance and quality-control pathways while preserving systemic stability.

### 4.2. Inflammasome-Related Transcriptional Changes Without Systemic Cytokine Escalation

A central observation of this study is the dissociation between inflammasome-related transcriptional changes and circulating cytokine output. Although NLRP3 and IL1B transcripts increased during fasting, circulating IL-1β and IL-6 protein levels remained stable, while TNFα concentrations decreased.

Inflammasome activation classically involves a two-step process consisting of transcriptional priming followed by activation-dependent assembly and cytokine maturation [14,15]. The absence of systemic cytokine elevation despite increased transcript levels suggests that fasting is associated with increased transcriptional activity of inflammasome-related genes at the mRNA level, without evidence of downstream inflammatory activation. This pattern is consistent with increased transcriptional responsiveness with potentially limited functional activation, although direct evidence for inflammasome activation is not available in the present study [16]. Therefore, interpretations regarding functional inflammation activity remain indirect.

Several mechanisms may contribute to this dissociation. Fasting is known to influence mTOR signaling and global translational control, potentially limiting protein synthesis despite increased mRNA availability [17]. In addition, inflammasome activation requires secondary activation signals, including complex assembly and caspase-1–mediated cytokine processing, which were not assessed in the present study [18]. The absence of increased circulating IL-1β or IL-6 may therefore reflect translational restraint or lack of downstream inflammasome activation rather than absence of transcriptional responsiveness.

Moreover, the significant reduction in TNFα protein levels further supports reduced systemic inflammatory signaling during fasting, consistent with TNFα as a key mediator of pro-inflammatory responses [19].

### 4.3. Baseline-Dependent Scaling of Inflammasome Responsiveness

One of the most notable findings of this analysis is the inverse association between baseline NLRP3 expression and ΔNLRP3 during fasting. Individuals with higher baseline expression exhibited attenuated transcriptional responses, whereas those with lower baseline expression showed greater induction. This baseline-dependent scaling explained a substantial proportion of inter-individual variability in transcriptional responsiveness.

Although skeletal muscle mass showed an association with ΔNLRP3 in univariable analyses, this relationship was attenuated after adjustment for baseline NLRP3 expression, suggesting that pre-existing immune tone rather than body composition may have contributed more substantially to variability in inflammasome responsiveness. Moreover, ANCOVA-style sensitivity analyses attenuated the statistical significance of the association between baseline and post-fasting NLRP3 expression, and the effect did not remain statistically significant. Therefore, regression-to-the-mean effects cannot be excluded and may have contributed to the observed association.

Taken together, these findings should be interpreted cautiously and considered exploratory. The observed directional trend across analyses is consistent with a potential biological constraint on transcriptional responsiveness, although its magnitude and robustness remain uncertain.

These findings suggest that fasting acts as a graded immunometabolic modulator, with response magnitude varying according to pre-existing immune tone.

This pattern is consistent with general principles of physiological homeostasis, which describe regulatory mechanisms that limit excessive system activation and maintain dynamic stability [20]. From a broader perspective, these results highlight baseline immune state as an important contributor to inter-individual variability in responses to metabolic stress, rather than a deterministic factor.

### 4.4. Inter-Individual Variability Reflects a Continuum Rather than Subtypes

Exploratory clustering suggested heterogeneity in Δ(T3–T1) molecular responses; however, structural separation between clusters was weak (mean silhouette width 0.12). Furthermore, cluster membership did not independently predict ΔNLRP3 after adjustment for baseline expression.

Collectively, these findings do not provide strong evidence for discrete molecular response subtypes and instead suggest that fasting-induced responses may follow a continuous spectrum of inter-individual variability. This observation should be interpreted cautiously, given the limited sample size and exploratory nature of the clustering approach. This observation further reinforces the role of baseline immune tone in shaping the magnitude of transcriptional adaptation.

### 4.5. Integration with Prior Work

Previous studies from this Ramadan cohort demonstrated activation of the autophagy pathway, modulation of inflammatory mediators without systemic cytokine escalation, and preservation of clinical stability during prolonged fasting. The present integrative analysis extends these observations by linking transcriptional changes to circulating protein measurement and identifying baseline-dependent scaling as a key determinant of immune responsiveness within this exploratory framework.

By integrating longitudinal gene expression data, circulating cytokine profiles, and statistical modeling approaches, this study provides a multidimensional exploratory characterization of fasting-associated immunometabolic remodeling.

### 4.6. Limitations

This study has several limitations. First, the analysis was conducted in a relatively small cohort of healthy young males, which may limit generalizability to other populations, including women, older individuals, or participants with chronic inflammatory conditions. Second, functional inflammasome activation was not directly assessed, as measurements of caspase-1 activity or cytokine maturation were not performed in this dataset. Consequently, conclusions regarding inflammasome activation remain inferential and based on transcriptional and circulating protein data.

Third, transcriptional changes do not necessarily reflect protein-level dynamics at the cellular level, and further studies incorporating multi-omics or functional assays are warranted. Fourth, potential regression-to-the-mean effects may contribute to the observed baseline-dependent associations between baseline NLRP3 expression and ΔNLRP3. Although an ANCOVA-based sensitivity analysis was performed, the association was attenuated and did not remain statistically significant. Therefore, this finding should be considered exploratory and requires confirmation in larger prospective studies.

Fifth, missing data were handled using complete-case analysis for each statistical test, and no imputation was performed due to the exploratory nature of this secondary analysis and the limited sample size. Sixth, although baseline characteristics were comparable, ethnic distribution differed significantly between the fasting and non-fasting groups (*p* < 0.001), reflecting recruitment of the original cohort. As molecular analyses were restricted to within-subject comparisons in the fasting group, ethnicity was not included as a covariate; however, this imbalance may still affect between-group descriptive interpretation. Seventh, variables known to change during Ramadan fasting, including sleep patterns, circadian rhythms, hydration status, physical activity, and dietary composition, were not systematically monitored in the present secondary analysis. These factors may influence gene-expression profiles and circulating cytokine concentrations and therefore represent potential sources of residual confounding.

Finally, as a secondary and exploratory analysis of the existing dataset, the findings should be interpreted as hypothesis-generating and not as evidence of causality or mechanistic confirmation.

## 5. Conclusions

Prolonged fasting is associated with coordinated immunometabolic changes characterized by autophagy activation, modulation of stress-associated signaling, and inflammasome-related transcriptional changes consistent with a primed-like state at the transcription level, in the absence of systemic inflammatory escalation. Importantly, inflammasome responsiveness appears to be baseline-dependent, suggesting that fasting may be associated with graded immunometabolic responses rather than a uniform pro-inflammatory response.

These findings support a model of transcriptional readiness with preserved systemic restraint and highlight the central role of baseline immune tone in shaping adaptive responses to metabolic stress. This observed dissociation between increased inflammasome-related mRNA expression and stable circulating cytokine levels further suggests the presence of post-transcriptional regulatory mechanisms that constrain inflammatory output.

Collectively, this work underscores the heterogeneous nature of immune responses to fasting and suggests that baseline-informed considerations may be relevant when interpreting individual variability in fasting responses.

## Figures and Tables

**Table 1:** Baseline demographic and anthropometric characteristics of participants prior to Ramadan fasting *.

Characteristic	Control (Non-Fasting) *n* = 25 ^1^	Fasting *n* = 25 ^1^	*p*-Value ^2^
Age (years)	26.2 ± 4.9	26.1 ± 4.9	>0.9
BMI (kg/m^2^)	24.6 ± 3.9	24.8 ± 3.6	0.8
Body weight (kg)	76.2 ± 21.4	77.8 ± 12.3	0.7
Body fat mass (kg)	17.4 ± 8.4	16.7 ± 7.4	0.7
Skeletal muscle mass (kg)	34.6 ± 7.4	34.7 ± 5.1	>0.9
Ethnic background			<0.001
Asian	9 (36%)	4 (16%)	
Europe	11 (44%)	3 (12%)	
Mid East	5 (20%)	18 (72%)	

* Continuous variables are presented as mean ± SD and categorical variables as *n* (%). Between-group comparisons were performed using Welch’s *t*-test and Fisher’s exact test, as appropriate. Ethnic distribution differed between groups due to recruitment characteristics of the original cohort and was not included as a covariate in downstream within-group analyses. ^1^ Mean ± SD for continuous variables; n (%) for categorical variables. ^2^ Welch’s two-sample *t*-test or Fisher’s exact test, as appropriate.

**Table 2:** Clinical and anthropometric changes during Ramadan fasting in the fasting group (ΔT3–T1) **.

Variable	*n*	Δ (mean ± SD)	95% CI	FDR
Body weight (kg)	25	−1.78 ± 1.44	−2.38 to −1.19	<0.001
BMI (kg/m^2^)	25	−0.56 ± 0.47	−0.75 to −0.37	<0.001
Skeletal muscle mass (kg)	25	−0.72 ± 1.22	−1.22 to −0.22	0.018
Fat-free mass (kg)	25	−1.07 ± 1.93	−1.86 to −0.27	0.021
Body water mass (kg)	25	−2.86 ± 7.52	−5.96 to 0.25	0.112
Systolic blood pressure (mmHg)	25	−3.56 ± 10.55	−7.91 to 0.79	0.139
Diastolic blood pressure (mmHg)	25	0.84 ± 9.72	−3.17 to 4.85	0.669
Body fat mass (kg)	25	0.64 ± 6.93	−2.22 to 3.50	0.669

** Values represent mean change ± SD from baseline (T1) to end of fasting (T3; ~30 days). Analyses were performed within the fasting group using paired comparison. Confidence intervals represent paired mean differences. False discovery rate (FDR) was adjusted using the Benjamini–Hochberg procedure within the clinical variable set.

## Data Availability

The data supporting the findings of this study are available from the corresponding author upon reasonable request. The data are not publicly available due to privacy and ethical restrictions involving human participants.

## Supplementary Materials

The following supporting information can be downloaded at https://www.mdpi.com/article/10.3390/nu18121954/s1, Figure S1: Longitudinal mRNA expression trajectories during fasting (T1–T4); Figure S2: Unsupervised clustering of Δ(T3–T1) transcriptional responses in the fasting group; Table S1: Changes in mRNA transcripts and circulating proteins during Ramadan fasting in the fasting group (ΔT3–T1).

## Abbreviations

The following abbreviations are used in this manuscript:

AI	Artificial Intelligence
ASC	Apoptosis-associated speck-like protein containing a CARD
ATG5	Autophagy Related 5
BECN1	Beclin 1
BDNF	Brain-Derived Neurotrophic Factor
BFM	Body Fat Mass
BFP	Body Fat Percentage
BMR	Basal Metabolic Rate
BW	Body Weight
BWM	Body Water Mass
cDNA	Complementary DNA
DRKS	German Clinical Trials Register
ELISA	Enzyme-Linked Immunosorbent Assay
FDR	False Discovery Rate
FFM	Fat-Free Mass
FG	Fasting Group
GAPDH	Glyceraldehyde-3-Phosphate Dehydrogenase
GDNF	Glial Cell Line-Derived Neurotrophic Factor
IGF-1	Insulin-Like Growth Factor 1
IL	Interleukin
IL1B	Interleukin-1 Beta (gene)
IL-1β	Interleukin-1 Beta (protein)
IL-6	Interleukin-6
IL-8	Interleukin-8
IL-10	Interleukin-10
IL-12	Interleukin-12
MMP-9	Matrix Metalloproteinase 9
mRNA	Messenger Ribonucleic Acid
NLRP3	NOD-, LRR-, and Pyrin Domain-Containing Protein 3
NEB	New England Biolabs
p16INK4A	Cyclin-Dependent Kinase Inhibitor 2A
p21	Cyclin-Dependent Kinase Inhibitor 1A
p53	Tumor Protein p53
qPCR	Quantitative Polymerase Chain Reaction
RNA	Ribonucleic Acid
SD	Standard Deviation
SE	Standard Error
SMM	Skeletal Muscle Mass
T1	Baseline (1 Week Pre-Ramadan)
T2	Mid-Ramadan
T3	End of Ramadan (~30 Days)
T4	1 Week Post-Ramadan
TNFA	Tumor Necrosis Factor Alpha (gene)
TNFα	Tumor Necrosis Factor Alpha (protein)
ULK1	Unc-51 Like Autophagy Activating Kinase 1
Δ(T3–T1)	Change from Baseline to End of Fasting
