# Supplementary figures and images for: Baseline-Dependent Immunometabolic Responses During Prolonged Intermittent Fasting: A Secondary Integrative Analysis
Source: Nutrients. 2026 Jun 17;18(12):1954. doi: 10.3390/nu18121954 (PMC13304944; doi:10.3390/nu18121954)

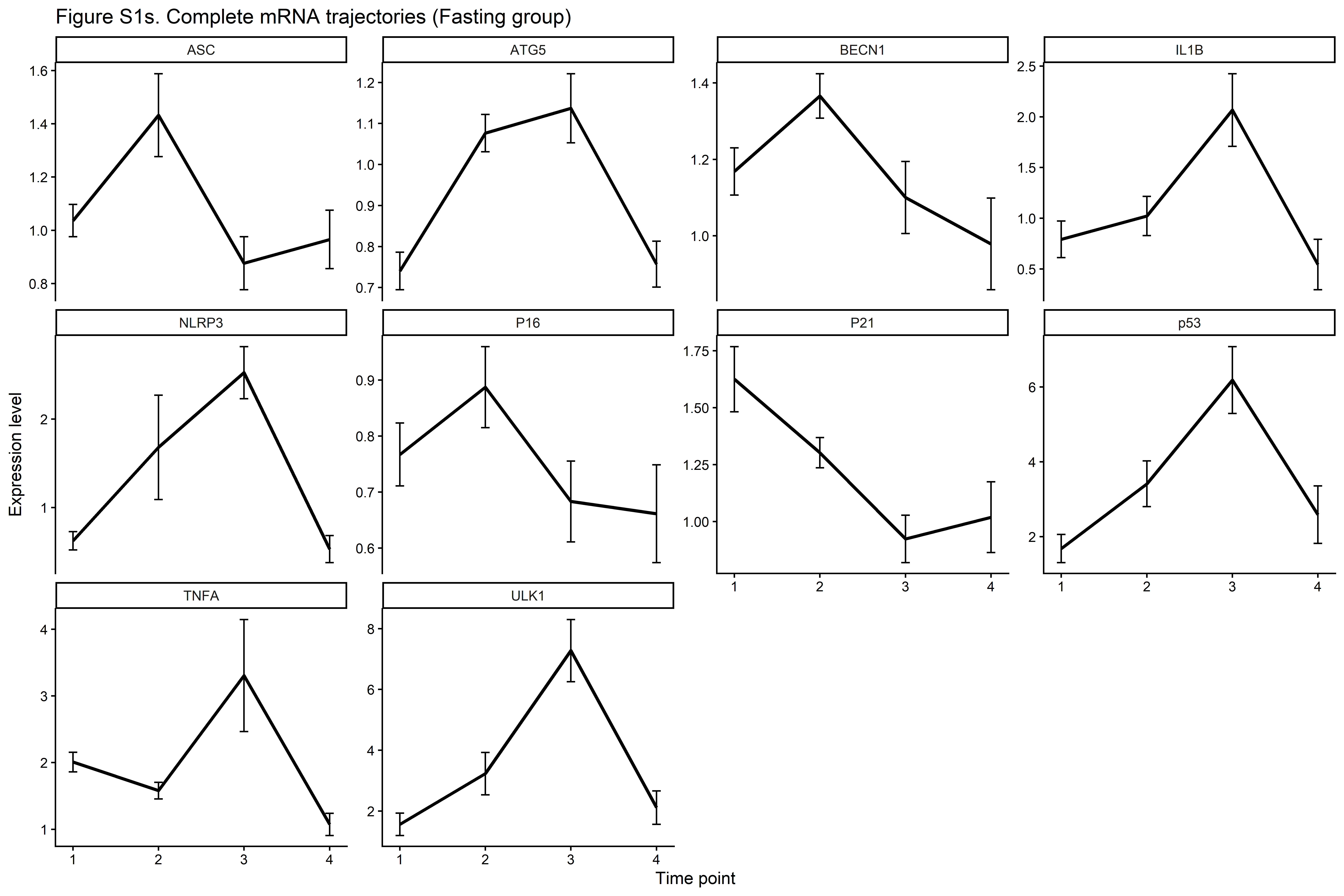

Supplement: Supplementary file 1 [file nutrients-18-01954-s001.zip › Figure_S1_mRNA_Trajectories.tiff]

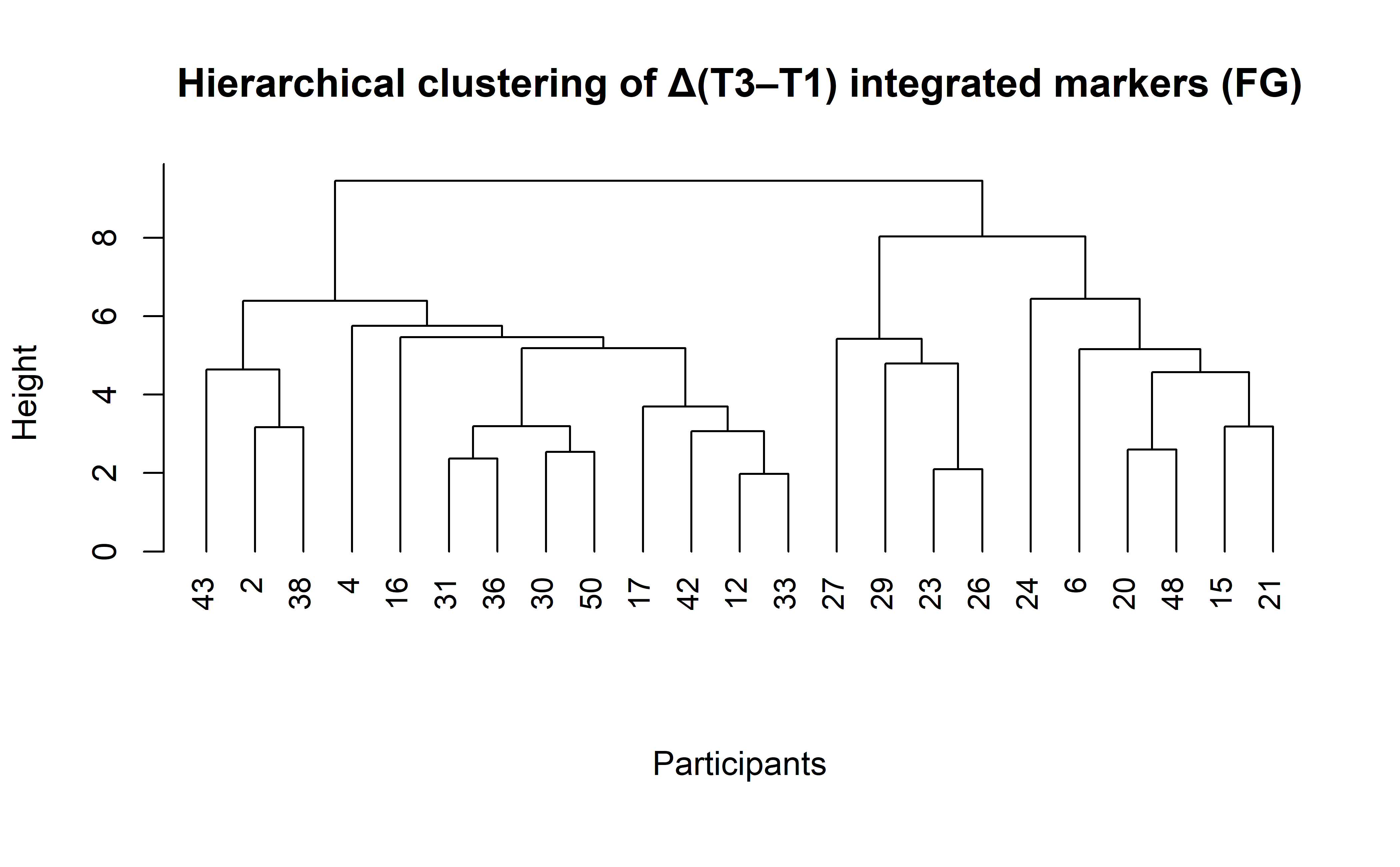

Supplement: Supplementary file 1 [file nutrients-18-01954-s001.zip › Figure_S2_Cluster_Dendrogram.png]
